# A critical review of recent developments in radiotherapy for non-small cell lung cancer

**DOI:** 10.1186/s13014-016-0693-8

**Published:** 2016-09-06

**Authors:** Sarah Baker, Max Dahele, Frank J. Lagerwaard, Suresh Senan

**Affiliations:** 1 Department of Radiation Oncology, Cross Cancer Institute, 11560 University Avenue, Edmonton, AB Canada T6G 1Z2; 2 Department of Radiation Oncology, VU University Medical Center, De Boelelaan 1117, Postbox 7057, 1007 MD Amsterdam, The Netherlands

**Keywords:** Radiotherapy, Non-small cell lung cancer, Intensity-modulated radiotherapy, Stereotactic ablative radiotherapy, Proton therapy

## Abstract

Lung cancer is the leading cause of cancer mortality, and radiotherapy plays a key role in both curative and palliative treatments for this disease. Recent advances include stereotactic ablative radiotherapy (SABR), which is now established as a curative-intent treatment option for patients with peripheral early-stage NSCLC who are medically inoperable, or at high risk for surgical complications. Improved delivery techniques have facilitated studies evaluating the role of SABR in oligometastatic NSCLC, and encouraged the use of high-technology radiotherapy in some palliative settings. Although outcomes in locally advanced NSCLC remain disappointing for many patients, future progress may come about from an improved understanding of disease biology and the development of radiotherapy approaches that further reduce normal tissue irradiation. At the moment, the benefits, if any, of radiotherapy technologies such as proton beam therapy remain unproven. This paper provides a critical review of selected aspects of modern radiotherapy for lung cancer, highlights the current limitations in our understanding and treatment approaches, and discuss future treatment strategies for NSCLC.

## Background

Lung cancer is the most frequently diagnosed cancer worldwide and the leading cause of cancer mortality, accounting for over 1.6 million deaths annually [1]. The role of curative-intent radiotherapy (RT) is well established in locally advanced [2] and early stage [3] non-small cell lung cancer (NSCLC). Nonetheless, the thorax remains a challenging anatomical site for RT delivery, due to the low electron density of lung, respiratory- and cardiac-induced tumor motion, and proximity of critical structures such as the esophagus and spinal cord. While advanced RT technologies can address many of these challenges [4–7], in most cases, the clinical benefit of such technology still needs to be demonstrated, especially since radiation oncology was the medical specialty generating the greatest increase in Medicare expenditures between 2003 and 2009 [8]. However, the evaluation of new technologies remains challenging. This review will discuss the current state of modern RT for NSCLC, limitations, and strategies to improve clinical outcomes in the future.

### Early stage, localized disease: lung SABR

The impact of advanced RT technology is perhaps most evident in the setting of early-stage NSCLC. Stereotactic ablative radiotherapy (SABR) is now considered the standard of care for medically inoperable patients with peripheral early-stage NSCLC [3]. SABR utilizes small margins for positional uncertainty, facilitated by 4-dimensional computed tomography (4DCT), multiple conformal or intensity modulated beams or arcs and volumetric image-guidance [9]. While peripheral lung SABR can also be delivered without these technologies, newer techniques can increase treatment efficiency and user confidence. Treatment-related toxicity with peripheral lung SABR is modest [10–12]. As SABR is not universally available, it is reassuring that data from the randomized SPACE study in patients with peripheral NSCLC suggest similar tumor outcomes with conventionally fractionated 3-dimensional conformal radiotherapy to 70 Gy [13].

There is an ongoing debate about the role of SABR in patients who are fit to undergo surgery [9]. A pooled analysis of two randomized trials of operable patients which closed prematurely due to slow accrual, showed a 16 % higher 3-year survival with SABR compared to surgery (*p* = 0.037). This was due to the higher rate of peri-operative mortality in the surgical group [14]. A propensity score matched analysis revealed that rates of treatment associated mortality and severe toxicity were lower with SABR for stage I-II NSCLC than with lobectomy performed by minimally-invasive video-assisted thoracoscopic surgery (VATS) [15]. Data from both retrospective [16, 17] and prospective phase II studies of SABR suggest survival outcomes similar to surgery [12, 18]. Shared decision-making tools may assist operable patients and their clinicians to arrive at a management plan based on a patient’s preferences and values [19, 20]. The role of SABR in surgical patients continues to be examined in 3 studies (NCT02468024, NCT02629458, NCT01753414), with a fourth (VALOR study) due to open this year. Both the SABRTooth and STABLE-MATES trials focus on high-risk patients.

Further improvements in SABR outcomes may come from strategies to reduce the rates of local-regional and distant failure, and from technology improvements that facilitate SABR in challenging scenarios such as central tumors (Table 1).

**Table 1:** Challenges and solutions for difficult SABR scenarios

Clinical scenario		Challenges	Potential solutions being explored
Pre Treatment	Incorporating patient preferences for treatment	Choice of SABR in operable NSCLC	• Shared decision-making [19, 20] • Comparative effectiveness research (including patient-reported outcomes, QOL and cost-effectiveness analyses) with “big data” strategies to facilitate data mining • RCTs underway (NCT02629458, NCT01753414, NCT02468024, VALOR study)
	Obtaining a diagnosis	Risks of treating benign disease Risks of biopsy in frail patients	•Use validated models for cancer risk determination in a given population [9] • Explore blood biomarkers [123]
Treatment	Central tumors Multiple primary lung cancers	Proximity to OARs Uncertainty in OAR location Uncertainly in OAR dose constraints	• “Big data” strategies to establish more reliable OAR dose constraints • MRI-guided adaptive RT [44] • Protons [41]
	Oligometastases	Higher pneumonitis risk Identify molecular and clinical characteristics of patients likely to benefit from ablative local therapies Optimize sequencing of RT and new systemic treatments	• Phase I-II trials, as well as randomized trials
Follow-up	Detection of recurrences	Distinguishing post-RT fibrosis vs recurrent disease	• Radiomic approaches [24]
	Survivorship issues	Loco-regional recurrences and second lung tumors Smoking cessation	• Survivorship clinics [124] • Patient-reported outcomes, including financial impact of treatments

Abbreviations *QOL* quality of life, *RT* radiotherapy, *SABR* stereotactic ablative radiotherapy, *NSCLC* non-small cell lung cancer, *OAR* organ at risk, *PTV* planning target volume

### Recurrences

Local failures following SABR include recurrences in the treated lesion or involved lobe, which are in the order of 9–20 % at 5 years [12, 16]. True rates of local control can be difficult to ascertain due to post treatment fibrosis, and radiologic changes can continue to evolve many years after treatment [21]. So-called ‘high-risk features’ on serial computed tomography (CT) scans may allow post-SABR fibrosis to be distinguished from local recurrence [22, 23] and image texture analysis merits investigation for the early identification of disease recurrence [24]. Radiological follow-up in accordance with ESMO guidelines may enable early identification of salvageable local/regional failures [25–27].

Regional lymph node failures have been observed in between 13–15 % of SABR patients at 5 years [12, 16] which appears comparable to lobectomy [15, 28, 29]. The role of routine endoscopic mediastinal and hilar nodal staging in patients without suspicious findings on positron emission tomography (PET)-CT studies is currently the subject of prospective studies [NCT01786590; NCT02719847]. When isolated hilar or mediastinal nodal failures occur, salvage radiotherapy may be possible in more than 50 % of patients, and appears well tolerated [30].

Approximately 20 % of patients develop distant disease recurrence following SABR [31, 32], which is once again similar to that observed after surgery. This suggests that systemic therapies could be of benefit in selected patients, although the recruitment of medically inoperable, elderly patients into studies exploring combined SABR and cytotoxic chemotherapy has proven to be challenging (NCT01300299).

### Central early-stage NSCLC

**Fig. 1:**
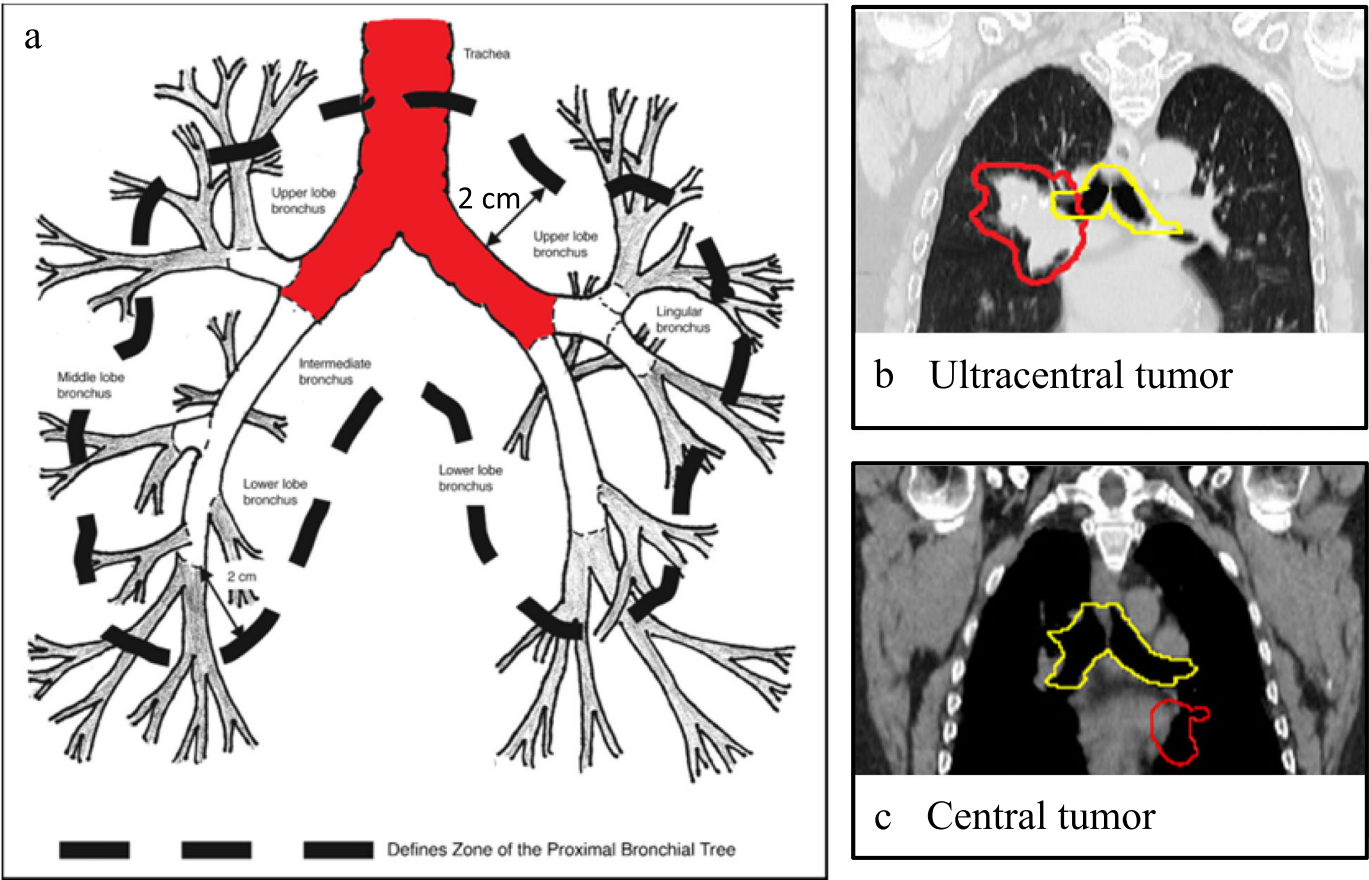
Definitions and examples of central and ultra-central lung tumors. **a** Diagram of the central airways of the lung. Reprinted with permission. ©2006. American Society of Clinical Oncology. All rights reserved. Timmerman, R et al.: J Clin Oncol 24(30), 2006: 4833–9. The black dashed line defines the location of tumors that are central relative to the proximal bronchial tree. The term central has been widened to include the region within 2 cm in all directions of any mediastinal critical structure, including the bronchial tree/trachea, esophagus, heart, brachial plexus, major vessels, spinal cord, phrenic nerve, and recurrent laryngeal nerve. The region shaded red shows the trachea and main bronchi, and lesions with a PTV which overlaps \this region are considered as ultracentral. **b** Example of an ultracentral tumor (planning target volume in red, and main bronchi/trachea in yellow). **c** Example of a central tumor

The Advanced Radiation Technology Committee of the International Association for the Study of Lung Cancer (IASLC) has defined ‘central tumors’ as those located within 2 cm in all directions of any mediastinal critical structure, including the bronchial tree, esophagus, heart, brachial plexus, major vessels, spinal cord, phrenic nerve, and recurrent laryngeal nerve [33]. It is notable that severe toxicity was reported following delivery of SABR in 3 fractions to doses of 60–66 Gy to central tumors [34], but not when ‘risk-adapted’ dosing strategies were used [12]. Both a systematic review [35], and a recent update [36], suggest that risk-adapted SABR delivered in 8 fractions is an effective treatment for moderately central tumors. However, tumor location may help to explain some of the differences between reports. It is important to distinguish ‘moderately central’ tumors from lesions immediately adjacent to central airways, so-called ‘ultracentral lesions’ (Fig. 1). The latter term has been used to describe a PTV that overlaps the trachea or main bronchi [37], with increased toxicity reported for this subgroup after both conventional and hypo-fractionated radiotherapy schemes [37–39]. A retrospective study reported that likely or possibly treatment-related deaths occurred in 7.5 % of patients with moderately central tumors [36]. The recent Radiation Therapy Oncology Group (RTOG) 0813 trial aimed to establish the safest dose that can be delivered in 5 fractions for central lesions [40]. Preliminary data reported that patients treated with the highest dose level (60 Gy in 5 fractions) had a 23 % rate of grade 3–5 toxicity. It should be acknowledged that the true radiation tolerance for central organs at risk (OARs) remains unknown, and uncertainty in tumor and OAR positions during treatment adds to our inability to determine true cumulative doses.

**Fig. 2:**
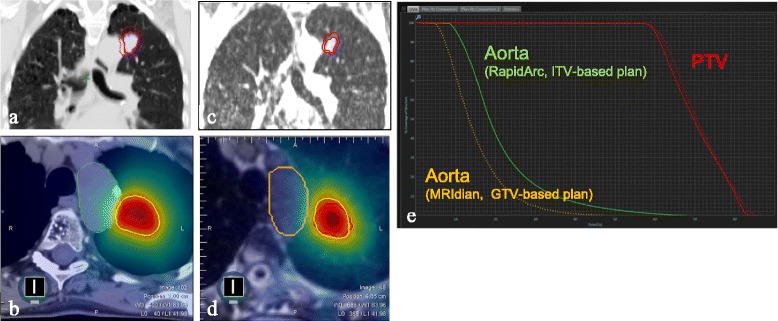
Comparative treatment plans for MRI-guided radiotherapy using breath-hold versus a standard free-breathing internal target volume (ITV)-based approach for a central tumor in a patient with interstitial lung disease. Panel **a** shows the ITV (7.8 cc) for a RapidArc (volumetric modulated arc therapy) plan, to which a 5 mm margin was added to derive a planning target volume (PTV, 26 cc); panel **b** the corresponding dose color-wash for an 8 fraction stereotactic ablative radiotherapy scheme to 60 Gy. Treatment was delivered using on-line MRI guided breath-hold on the MRIdian in which the target was the gross tumor volume (6.9 cc, Panel **c**), to which a 3 mm setup PTV margin was added (PTV 13.6 cc). Panel **d** shows the MRIdian dose color-wash, and Panel **e** the dose volume histograms for the adjacent aorta for both plans

It has been suggested that proton beam therapy (PBT) can allow for dose reduction to central structures [41], although the benefits of PBT may be questionable given its susceptibility to anatomic and positional variations [42]. Although on-line matched cone beam CT scans can be used to image OARs prior to irradiation [43], the field has been advanced by the recent introduction of magnetic resonance imaging (MRI) -guided RT delivery (MRIdian System, Viewray Inc, Cleveland, OH). The MRIdian platform facilitates online adaptive radiotherapy, and allows for direct tumor visualization during treatment delivery at 4 frames per seconds in the sagittal plane [44]. During gated radiotherapy using breath-hold mode, the system automatically shuts-off radiation delivery with a lag-time of 0.4 s (or less) when the target is outside pre-specified safety margins (Fig. 2). A number of other linac-MR delivery platforms are in development [45–47] and may contribute to advances in the practice of central SABR.

### Multiple primary lung cancers

The incidence of multiple synchronous primary lung cancers (MPLCs) can be as high as 4–8 % [48], and second primary lung cancers occur at a rate of approximately 3 % per year [27]. Several studies report excellent local control and modest toxicity following SABR for MPLCs [49–51]. As larger volumes of some OAR’s are irradiated in this situation, strategies designed to reduce tumor motion and dose to OARs are warranted.

### SABR and stage IV disease

In a randomized trial, surgical resection of a single brain metastasis combined with whole brain RT, more than doubled median survival from 15 to 40 weeks, and lengthened functional independence compared to RT alone [52]. More than three-quarters of patients in the study by Patchell et al. consisted of patients with NSCLC. In unselected oligometastatic patients, however, rates of progression-free survival (PFS) are highly variable, suggesting that many have more widely disseminated occult disease. In retrospective studies, rates of 5-year survival may approach 50 % in highly select patients, namely those with metachronous lesions, lower number of metastases and a good performance status [53]. A recent multi-centre phase II trial randomized NSCLC patients with ≤ 3 metastases who did not progress after first line systemic treatment to either local consolidative therapy (surgery, RT or chemo-RT to all metastases, with or without systemic therapy) or to systemic therapy alone [54]. The study was closed early after only 49 patients were enrolled when interim analysis found the median PFS in the consolidative therapy arm to be 14.4 months compared to 3.9 months in standard arm. Although these findings are provocative, the limited patient numbers mean that additional studies will be required. The interest in exploring ablative treatments for oligometastatic disease will increase following the proposed revision in the 8th Edition of the TNM lung cancer classification system, where the current M1b category is subdivided into a new M1b, comprising a single extra-thoracic metastasis in a single organ, and M1c, encompassing multiple extra-thoracic metastases [55].

Another area of investigation is the use of SABR in the setting of oligo-progression, where disease that has initially responded to systemic treatment, subsequently demonstrates limited progression [56]. In patients with stage IV disease who receive molecular targeted therapy for an activating mutation of the EGFR receptor, or an ALK-translocation, and who subsequently develop progression at limited sites, the use of local ablative therapies is now recommended in the European Society for Medical Oncology (ESMO) guidelines [57].

### Locally advanced NSCLC

Stage III NSCLC remains a challenging disease to treat. In randomized trials, the addition of surgery has not been shown to be of benefit to overall survival (OS), compared to definitive concurrent chemoradiotherapy (CRT) (Table 2). In a phase III trial of concurrent CRT, radiation dose escalation to 74 Gy had a detrimental effect on survival [58]. Rates of local and distant failure after CRT have remained constant over time (approximately 30–40 and 40–50 %, respectively) however median OS has improved modestly, by approximately 10 months (Table 3). The reasons for this improvement in OS are uncertain, but stage migration due to improved imaging may be one contributory factor [59]. In addition, the incidence of high-grade radiation pneumonitis and esophagitis has decreased significantly in the past decade [60]. Survival improvements may also reflect the availability of effective systemic therapies for the 50 % of patients who relapse with systemic disease [61], although the use of such therapies is not routinely captured in trials.

**Table 2:** Outcomes from randomized trials with a surgical arm in stage III non-small cell lung cancer

Trial	Inclusion	Staging PET or PET/CT	Study question	RT^a^	Chemotherapy	N (randomized)	Answer	Treatment related mortality	5-year OS
EORTC 08941 [125]	Unresectable IIIA (N2)	Not mandatory	CT-S vs CT-RT	60–62.5 Gy to primary and involved mediastinum; 40–46 Gy to uninvolved mediastinum	Platinum-based with at least one other agent	332	No significant difference	4 % within 30 days of surgery 1 patient died of RP, timing NR	16 % 14 %
INT 0139^b^ [126]	Potentially resectable IIIA (N2)	Not mandatory	CRT-S vs CRT	45 Gy in CRT-S arm 61 Gy in CRT arm	Cisplatin-etoposide	429 (396 eligible)	No significant difference	8 % 2 % (No deaths during induction)	27 % 20 %
ESPATUE^c^ [127]	Resectable IIIA (N2) and selected IIIB	97 %	CT-CRT-S vs CT-CRT-CRTboost	Both arms: induction 45 Gy delivered as 1.5 Gy BID In definitive CRT arm: risk-adapted CRTboost to 65–71 Gy	Induction: cisplatin-paclitaxel Concurrent: cisplatin-vinorelbine	161	No significant difference, but closed early and was under- powered with respect to the primary end-point of OS	6 % in surgical arm 3 % in definitive CRT arm (2 additional patients died during induction)	44 % 40 %
SAKK 16/00 [128]	Resectable IIIA (N2)	Required (rate NR)	CT-RT-S vs CT-S	44 Gy (in 22 fractions over 3 weeks)	Cisplatin-docetaxel	232	No difference	0 % within 30 days of surgery 3 % within 30 days of surgery	40 % 34 %

Courtesy of Prof. Rafal Dziadziuszko. Discussant ESMO 2014 Madrid. Modified to update subsequent publication

*CT* induction chemotherapy, *CRT* concurrent chemoradiotherapy, *RT* radiotherapy; S surgery, *CRTboost* concurrent chemoradiotherapy boost, *RP* radiation pneumonitis, *NR* not reported, *BID* twice daily, *OS* overall survival

^a^RT doses in standard fractionation unless otherwise indicated

^b^Increased disease-free survival in surgery arm (12.8 vs 10.5 months; *p* = 0.017); unplanned analysis showed longer median OS in lobectomy subgroup vs matched CRT subgroup (33.6 vs 21.7 months; *p* = 0.002)

^c^246 enrolled (out of 500 planned). After induction treatment, patients with resectable tumors (*n* = 161, 65 %) randomized. In all 246 patients, 5 year OS 34 %

**Table 3:** Outcomes with definitive chemoradiotherapy for stage III non-small cell lung cancer

Trial	Inclusion	Staging PET-CT	Histology	Treatment regimen in standard CRT arm^a^	RT technique	N	PTV (mean)	Toxicity in standard CRT arm	Outcomes
RTOG 0617 [58]	Unresectable III	91 %	42/47 % squamous in 60/74 Gy arms	60 Gy Concurrent carboplatin-paclitaxel, followed by 2 cycles consolidation	46/47 % IMRT in 60/74 Gy arms (Remainder 3DCRT)	424 analyzable for radiation end-point	495/510 mL in the 60/74 Gy arm	In 60 Gy arm: Grade ≥ 3 RP 7 % Grade ≥ 3 esophagitis 7 % Grade 5 toxicity 3 %	In 60 Gy arm: Median OS 29 months 2-year OS 58 % 2-year LF 31 % 2-year DF 47 %
PROCLAIM [78]	Nonsquamous III	82 %	Non-squamous only	60–66 Gy Arm A: pemetrexed-cisplatin, pemetrexed consolidation Arm B: etoposide-cisplatin, non-pemetrexed consolidation	25 % IMRT (Remainder 3DCRT)	598	607/585 mL	Grade ≥ 3 RP 1.8/2.6 % Grade ≥ 3 esophagitis 15.5/20.6 % Grade 5 toxicity 1.7/1 %	Median OS 27/25 months Median PFS 11.4/9.8 months IFF (site of 1^st^ failure) 42 % DF (site of 1^st^ failure) 48 %
KCSG-LU05-04 [79]	Unresectable III	92 %	32 % squamous	66 Gy Concurrent docetaxel-cisplatin Arm A: CRT-observation Arm B: CRT-docetaxel-cisplatin consolidation	NR	437 eligible	NR	Grade ≥ 3 RP 1.2 % Grade ≥ 3 esophagitis 9.5 % Grade 5 toxicity 3.6 % during CRT, 2.9 % during consolidation	Median OS 20.6/21.8 months Median PFS 8.1/9.1 months After median follow-up time of 51 months: DF 25 % LRR 25 % DF and LF 3 %
RTOG 9410 [129]	Inoperable stage II-III	0 %	38 % squamous	63 Gy Cisplatin-Vinblastine	2DRT	610	N/A	For CRT with early RT arm: Grade ≥ 3 esophagitis 22 % Grade ≥ 3 acute RP 4 % Grade 5 toxicity 2 % (as worst overall toxicity)	For CRT with early RT arm: 5-year OS 16 % Median OS 17 months IFF only 25 % Out of field only 37 % Both IFF and out of field 10 %
Meta-analysis of 6 trials comparing CRT vs sequential CT/RT [130]	Unresected stage III	0 %	46 %	60 Gy (2 trials), 66 Gy, (1 trial), 66 Gy in 24 fractions (1 trial), 56 Gy split course (1 trial), 48.5 Gy (split course of 36 Gy in 12 fractions, 7 days rest, 12.5 Gy in 5 fractions) Single agent low-dose cisplatin (2 trials), cisplatin-based doublet (3 trials), carboplatin (1 trials)	3DCRT in 1 trial Remainder 2DRT	603/602 in concurrent/sequential groups	N/A	Grade ≥ 3 esophagitis 18 % (concurrent CRT) Rates of acute RP and Grade 5 toxicity NR	For concurrent CRT patients: 3-year OS 24 % 5-year OS 15 % 3-year LRF 28 % 5-year LRF 29 % 3-year DF 40 % 5-year DF 41 %

Abbreviations *CRT* chemoradiotherapy, *CT* chemotherapy, *RT* radiotherapy, *IMRT* intensity modulated radiotherapy, *PTV* planning target volume, *mL* milliliters, *N/A* not applicable, *RP* radiation pneumonitis, *OS* overall survival, *DFS* disease free survival, *IFF* in-field failure, *LF* local failure, *DF* distant failure, *LRR* locoregional recurrence, *NR* not reported, *3DCRT* three﻿- ﻿dimensional conformal radiotherapy, *2DRT* two-dimensional radiotherapy

^a^All RT standard fractionation

Currently, ESMO recommends conventionally fractionated CRT to 60–66 Gy, with two to four concomitant cycles of chemotherapy to treat locally advanced NSCLC, with no evidence for induction or consolidation chemotherapy [2]. In patients unfit for concurrent CRT, accelerated RT delivery is suggested. In practice, significant numbers of patients are not fit to undergo CRT; 20 % or more of patients with stage IIIA receive only palliative treatment, with another 12 % receiving RT as a single modality [62]. In patients eligible only for RT, image-guided hypofractionated RT is a strategy that merits investigation, although it should be acknowledged that competing causes of mortality in such patients may limit major improvements in OS.

### Post-operative RT

The role of post-operative RT (PORT) in patients with completely resected N2 disease remains unclear [63]. An earlier meta-analysis using older radiotherapy techniques failed to show a survival benefit for this patient group [64]. More recent population studies have suggested a survival benefit with PORT for pN2 disease [65, 66]. However, pre-operative mediastinal lymph node staging has improved significantly in the past decade, with the use of FDG-PET scans and endoscopic staging, resulting in N2 disease that is discovered only at the time of surgery being a less common scenario. Definitive conclusions of the role of PORT in N2 disease must await the results of an ongoing phase III trial, in which both surgical procedures and RT techniques are clearly specified (LungART, NCT00410683).

### Have newer RT technologies improved survival in stage III NSCLC?

A number of innovations in RT have been introduced in the past two decades [67]. The replacement of conventional treatment simulation with CT simulation has been associated with a survival advantage in the SEER population [6]. Guidelines now recommend 4DCT simulation, and cone beam CT (CBCT) for image-guidance which has reduced planning target volume (PTV) margins [68]. More accurate dose calculation algorithms are in clinical use [4], and more conformal radiation delivery can be achieved with intensity-modulated RT (IMRT) and PBT [7, 69]. Improved OAR sparing with more conformal dose distributions, and on-line image-guidance, may have contributed to the approximately 10 % reduction in acute esophagitis rates seen in recent years (Table 2).

### Intensity-modulated RT

**Fig. 3:**
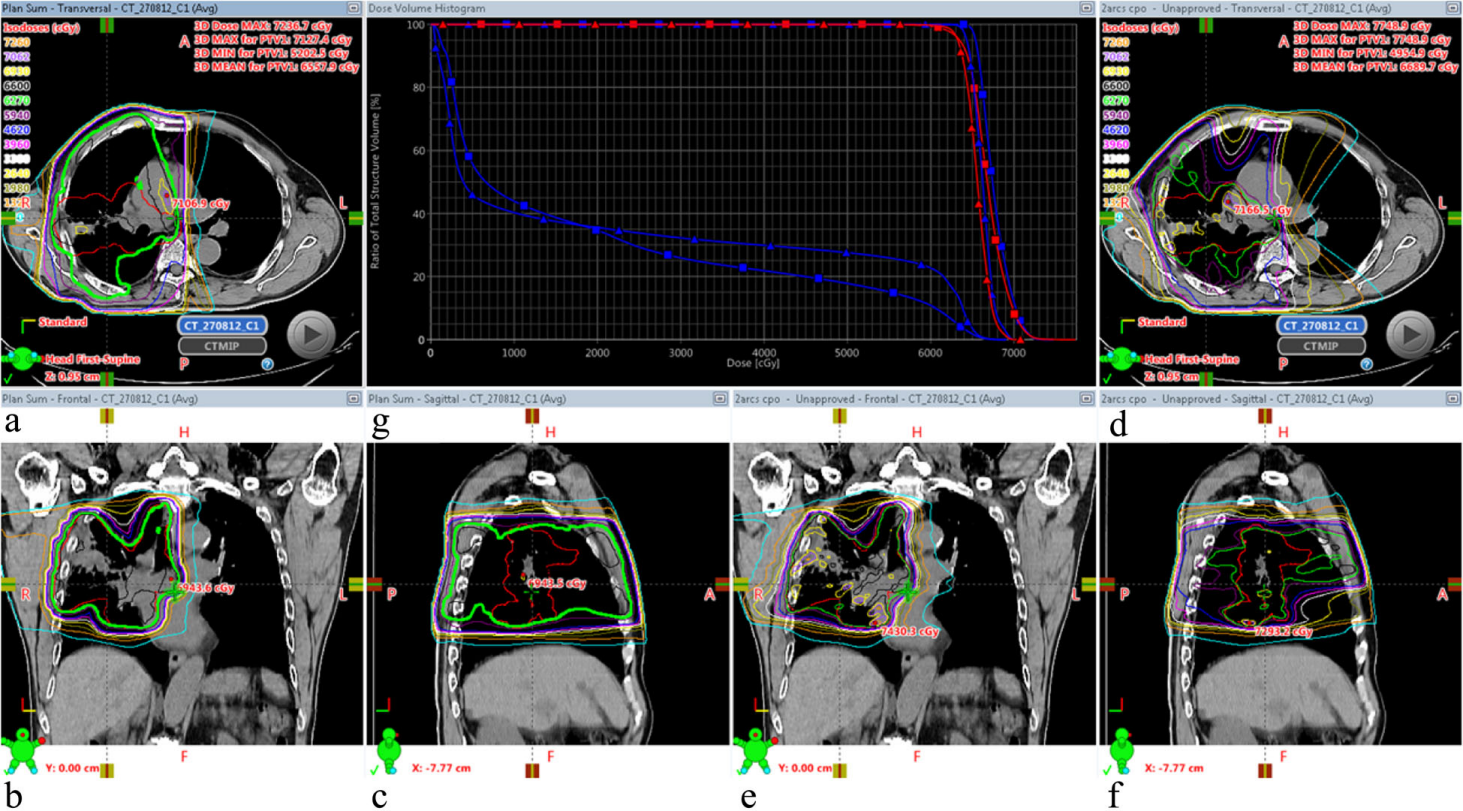
A comparison of two radiotherapy techniques delivering 66 Gy in 33 fractions to a locally-advanced lung tumor. Panels **a**-**c** show axial, coronal, and sagittal views of a hybrid-intensity-modulated radiotherapy (IMRT) plan; panels **d**-**f** show the corresponding views of a volumetric modulated arc therapy (VMAT) plan for the same tumor. Panel **g** shows the dose-volume histogram of the hybrid IMRT plan (*triangles*) and VMAT plan (*squares*); the red and blue lines to the right represent the planning target volume (PTV) and internal target volume (ITV) respectively; the remaining pair of blue lines represent the lung volume (lung tissue outside the PTV). PTV and ITV coverage is comparable for both techniques (**g**). The VMAT plan has a more conformal 95 % isodose (green line) around the PTV (**d**-**f** compared with **a**-**c**), however the maximum dose in the PTV is higher (**g**). The amount of lung receiving ≤20 Gy is very similar with both techniques (**g**), but the VMAT plan has a lower mean lung dose (19.5 Gy vs 22 Gy with hybrid-IMRT) and the hybrid-IMRT plan has more contralateral lung sparing, as seen by the position of low-dose isodose lines (orange [1320 cGy] and light blue [660 cGy])

Planning studies have consistently demonstrated gains with IMRT compared with 3- dimensional conformal RT (3DCRT), for metrics including mean lung dose, lung V20, spinal cord dose, and heart doses [7, 70]. However volumes of low-dose irradiation may increase with some IMRT delivery approaches [71] (Fig. 3). IMRT has been rapidly adopted for lung cancer despite a paucity of evidence [72]. A SEER analysis suggested that the main predictors of IMRT utilization were geographical location, and freestanding versus hospital-based center, rather than disease factors such as tumor size or stage [73]. Most comparisons of IMRT and 3DCRT for locally advanced NSCLC come from retrospective single-institution and registry-based analyses, all with well-recognized limitations. A National Cancer Data Base (NCDB) analysis found that the use of 3DCRT or IMRT improved survival in stage III patients, versus those treated with CRT using 2-dimensional RT(2DRT) [74]. However, when 3DCRT and IMRT were evaluated separately, there was no added survival with IMRT. Other analyses have also reported no survival or toxicity improvement with IMRT [73, 75, 76], although these studies were conducted across heterogeneous patient groups. It is possible that the gains from IMRT are limited to specific patient groups, and another NCDB analysis suggested improved median and 5-year survival with IMRT for T3 and T4 tumors [77]. Unfortunately, many databases lack the comprehensive clinical and dosimetric data necessary to study the nature of the relationship between technology and outcomes.

It is notable that in recent trials in which half or more of patients were treated with 3DCRT, the rates of grade ≥ 3 pneumonitis following doses of up to 66 Gy, were only in the range of 1.2–7 % [58, 78, 79]. Data from the recent RTOG 0617 dose escalation study merit closer inspection [58]. Approximately equal numbers of patients were treated with 3DCRT or IMRT contemporaneously, avoiding the confounding time factor present in retrospective analyses. Despite the IMRT group having a mean PTV about 15 % larger and more stage IIIB tumors, rates of grade ≥ 3 pneumonitis were reduced from 7.9 to 3.5 %. Furthermore, the IMRT cohort was more likely to receive full-dose consolidative chemotherapy [7], and reported less decline in quality of life at 12 months [80]. However, patients treated at higher accruing centers experienced a striking 10 % survival advantage at 2 years [81]. These centers had higher rates of IMRT utilization, which was not independently predictive of survival, raising the question of whether the benefits attributed to IMRT in earlier analyses were in fact due to other, unrecognized factors associated with treatment at high accruing centers. Although the heart V5 and V30 were reported as predictive of survival in RTOG 0617, the lung dose, a well-recognized predictor of severe toxicity, was not included in the multivariate analysis. A subsequent analysis in an independent cohort found mean lung dose, but not heart doses, to be predictive of survival; there was a strong correlation between mean heart dose and heart V5 with the mean lung dose [82].

A number of groups are investigating if the IMRT delivery of higher doses to tumor regions that show high or persistent ^18^F-flurodeoxyglucose (FDG)-PET uptake, will lead to improved survival [NCT01024829; NCT02788461; NCT01507428; NCT02790190]. A common underlying hypothesis for these trials is that local relapses may be more frequent in the high FDG uptake regions of primary tumors. Outcomes of the ongoing trials are awaited.

### Proton beam therapy

Facilities for PBT have grown rapidly in recent years, even though limited data exists for its cost-effectiveness in NSCLC [83, 84]. Highly conformal high dose distributions can theoretically be achieved, allowing for further reduction in doses to normal structures compared to IMRT [69, 85]. PBT is currently delivered either in passively scattered proton therapy (PSPT) mode, or pencil-beam scanning (PBS), which can deliver intensity-modulated proton therapy (IMPT). Planning studies have suggested that PBS can allow greater sparing of critical structures than PSPT [86, 87], but it may be more sensitive to changes in position or anatomy [41, 88].

A single-institution retrospective comparison of three treatment techniques (3DCRT, IMRT and PSPT) in locally advanced NSCLC, reported that proton delivery resulted in lower rates of grade 3 or higher pneumonitis and esophagitis (2 and 5 %, respectively; 3DCRT, 30 and 18 %; IMRT, 9 and 44 %; *p* < 0.01 for all) [89]. However, the rates of esophagitis are inconsistent with findings observed in recent phase III studies. A prospective randomized trial led by the MD Anderson Cancer Center compared photon IMRT versus PSPT, and reported no differences in treatment failures, which were defined as either grade ≥3 pneumonitis or local failure at 1 year [90, 91]. A second phase III trial with a target accrual of 560 stage II-IIIB NSCLC patients is now underway (RTOG 1308). Both PSPT and PBS are still permitted in this study. While the improved OAR sparing with PBT makes it a seemingly attractive option for treating large tumors, a large volume has consistently been associated with poorer survival [92–94], which suggests that survival gains may be modest, at best. There is, therefore, currently no high-level evidence to support the routine use of proton therapy in locally advanced NSCLC, and evidence supporting IMRT is based on population-based analysis of patient sub-groups. 3DCRT therefore remains an important treatment option, especially as access to radiotherapy is limited in many countries, and escalating costs are of concern [95, 96].

### Radiation and immunity

**Fig. 4:**
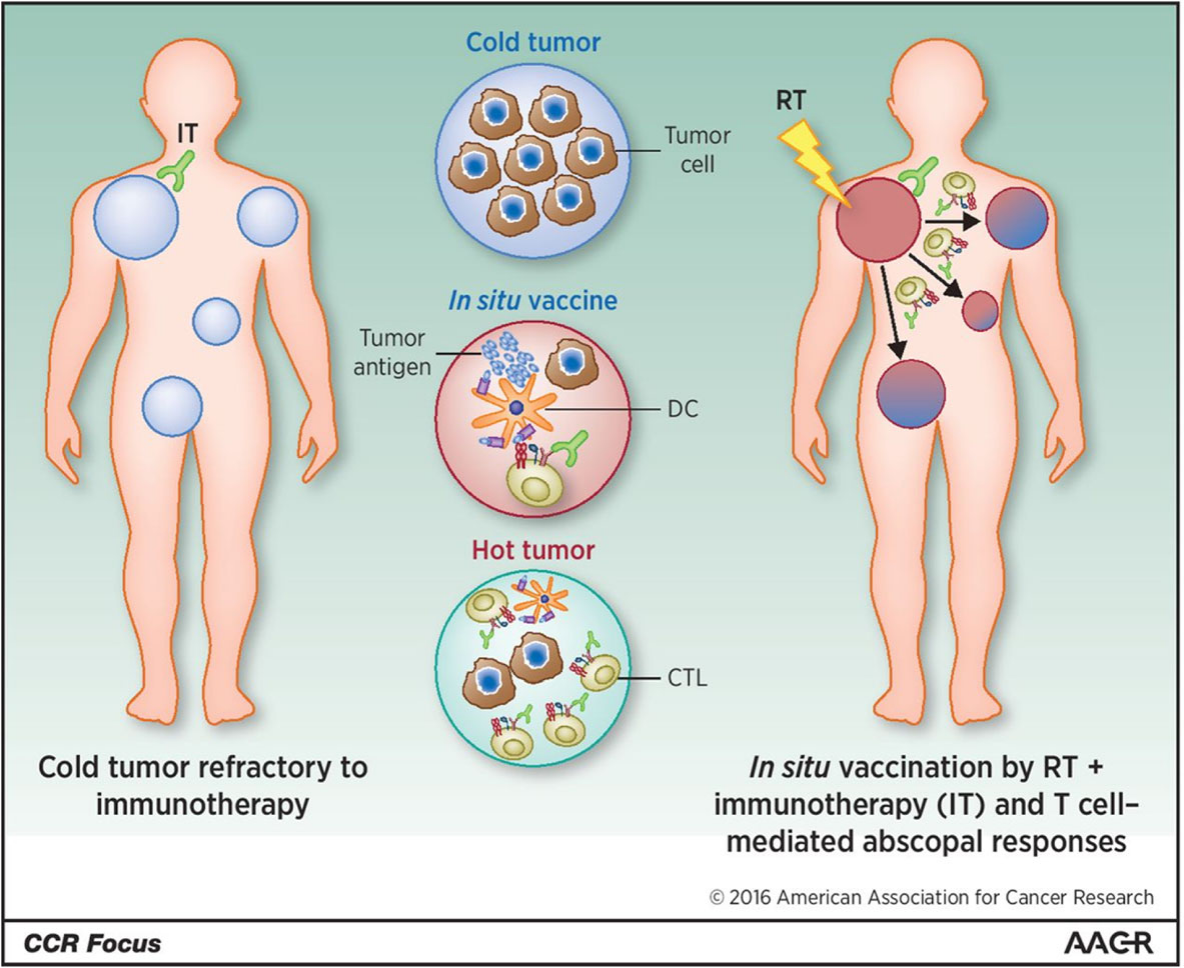
Reprinted with permission. Theresa L. Whiteside et al. Clin Cancer Res 2016;22:1845–1855. Schematic representation of immune-mediated abscopal effects. The systemic proinflamatory effects of irradiating a tumor mass results in it being ‘hot’, and acting as an ‘in situ tumor vaccine’ against distant non-irradiated tumors. Such a local response could be enhanced by administering immunostimulatory antibodies in order to attain an enhanced systemic effect, thereby exploiting the immune effects of radiotherapy. CTL, cytotoxic T cell; RT, radiotherapy

RT can have an immune stimulatory effect by generating tumor antigens, promoting a T-cell mediated anti-tumor response, and potentially causing immune-mediated abscopal effects where distant non-target lesions can regress [97] (Fig. 4). However, abscopal effects are very uncommon [98]. Radio-immunotherapy is a field of active research, and much remains unknown regarding the optimal sequencing of treatments, as well as optimal RT dose/fractionation schedules [99, 100]. Some data suggests that large doses per fraction used in SABR may be more effective, but the potential for unexpected toxicity exists, suggesting a need for careful treatment planning and delivery. More safety data will be forthcoming from ongoing clinical trials in this field [101].

### Challenges in evaluating new RT technologies

While classic RCTs remain the gold standard for generating evidence, their applicability for evaluating RT technology has been challenged [102, 103]. The high costs involved, the potential for a learning curve with new technology [104], and ethical concerns with a perceived lack of equipoise between older and new technologies, are all potential impediments. The extended duration of follow-up required to assess long-term toxicities precludes study completion in a timely manner, and by the time trial results are published, they may be considered invalid due to the interval evolution of technology.

In certain situations, comparative effectiveness research may be a more practical and financially feasible approach for evaluating treatments [105, 106]. Prospective multi-center registries provide access to large patient numbers and extensive data, which may be integrated and analyzed using a ‘big data’ approach [107]. Some authors have suggested that dosimetric/complication probability models may help identify patients most likely to benefit from advanced technologies [108], but there remains much uncertainty associated with such models [109]. Similarly, patient-reported outcomes (PROs) are being increasingly considered as important clinical endpoints, but PROs can be difficult to select and interpret as they may be influenced by diverse patient factors [110, 111]. The potential of PROs for evaluating radiotherapy research may be significant, as suggested by a mobile app interface for reporting patient-reported clinical symptoms in advanced NSCLC, that was shown to improve quality of life and survival [112].

By focusing on incremental improvements in technology, radiation oncologists may risk ignoring the fact that clinicians’ overall knowledge base and the patient’s health are often a more important determinant of patient outcome [113]. For example, a poor forced expiratory volume in one second (FEV1), and large gross tumor volumes, have been associated with a 3-fold increase in early mortality following CRT [114]. Interstitial lung abnormalities, as well as severe chronic obstructive pulmonary disease (COPD), are associated with high all-cause mortality [115, 116], and a higher risk of toxicity after CRT [117, 118]. Other patient factors, including weight loss during the first three weeks of CRT may also profoundly affect survival [119]. An improved understanding of what drives poor outcomes in patients with factors like large tumors and co-morbid illness is needed. If RT delivery is considered in isolation, measures such as the optimization of fractionation schedules for a given patient, or spatiotemporal optimization of radiation dose, are unlikely to result in large improvements in outcomes [120].

Furthermore, more accurate distinction between toxicity related to treatment versus symptoms related to comorbidities is needed. Common COPD symptoms which may be present in patients at baseline can easily be mislabeled as a grade 3 pulmonary toxicity. Simply correlating observed toxicities with OAR dose-volume parameters is insufficient, due to uncertainty in delivered dose [121, 122], and lack of anatomical and functional information. This means that more robust and comprehensive dosimetry reporting is needed in the future.

## Conclusion

Although innovations in treatment planning and delivery have led to more precise and accurate RT delivery, for the majority of NSCLC patients, further improvements in treatment outcomes are likely to come about from an integration of novel biological treatment strategies based on an understanding of cancer and radiotherapy at the molecular level. Understanding which patients may benefit most from a given RT technology, as well as identifying those who are at high risk of treatment toxicity, may help tailor the application of advanced technologies to those most likely to benefit and promote a personalized approach to lung cancer radiotherapy.

## Abbreviations

2DRT: Two- dimensional radiotherapy
3DCRT: Three- dimensional conformal radiotherapy
4DCT: Four- dimensional computed tomography
BED: Biologically effective dose
CBCT: Cone beam computed tomography
COPD: Chronic obstructive pulmonary disease
CRT: Concurrent chemoradiotherapy
CT: Computed tomography
FDA: United States food and drug administration
FDG-PET/CT: Flurodeoxyglucose-positron emission tomography/computed tomography
FEV1: Forced expiratory volume in 1 s
GTV: Gross tumor volume
HR: Hazard ratio
IMPT: Intensity-modulated proton therapy
IMRT: Intensity-modulated radiotherapy
ITV: Internal target volume
MPLCs: Multiple primary lung cancers
MRI: Magnetic resonance imaging
NCDB: National cancer database
NSCLC: non-small cell lung cancer
OARs: Organs at risk
OS: Overall survival
PBT: Proton beam therapy
PFS: Progression-free survival
PROs: Patient-reported outcomes
PSPT: Passively scattered proton therapy
PTV: Planning target volume
QALY: Quality-adjusted life-year
QOL: Quality of life
RCT: Randomized controlled trial
RT: Radiotherapy
RTOG: Radiation therapy oncology group
SABR: Stereotactic ablative radiotherapy
SEER: Surveillance, epidemiology, and end results
TKI: Tyrosine kinase inhibitor
VATS: Video-assisted thoracoscopic surgery
